# Abundance and Seasonal Migration Patterns of Green Lacewings (Neuroptera: Chrysopidae) across the Bohai Strait in Eastern Asia

**DOI:** 10.3390/insects15050321

**Published:** 2024-05-01

**Authors:** Xingya Wang, Haotian Ma, Yuechao Zhao, Ying Gao, Kongming Wu

**Affiliations:** 1 College of Plant Protection, Shenyang Agricultural University, Shenyang 110866, China; wangxingya20081@163.com (X.W.); mhtpony@163.com (H.M.); zhaoyuechao98512@163.com (Y.Z.); gy434585296@163.com (Y.G.); 2 State Key Laboratory for Biology of Plant Diseases and Insect Pests, Institute of Plant Protection, Chinese Academy of Agricultural Sciences, Beijing 100193, China

**Keywords:** green lacewing, biological control, migration route, migration trajectories, suction trap

## Abstract

**Simple Summary:**

Green lacewing is an important natural predator of many crop pests. In this study, we conducted 10 years of monitoring from 2012 to 2021 to determine the aerial migration patterns of green lacewing species using a suction trap on Beihuang Island (BH), a small island in the center of the Bohai Strait that serves as a seasonal insect migration pathway in eastern Asia. Overall, there were significant interannual and seasonal differences in the densities of the main species, and the sex ratio varied among different months, with more females being captured than males. It was determined that the green lacewings primarily originated from Shandong Province through trajectory analysis. This study provides valuable insights into the migration dynamics of green lacewings in eastern Asia, which is helpful for biological control in the region.

**Abstract:**

Many insects, including green lacewings, migrate seasonally to exploit suitable breeding and winter habitats. Green lacewings are important natural enemies of insect pests worldwide. Here, four dominant green lacewing species, *Chrysoperla nipponensis* (Okamoto), *Chrysopa pallens* (Rambur), *Chrysoperla furcifera* (Okamoto), and *Chrysopa formosa Brauer*, were investigated for their ability to migrate between northern and northeastern China across the Bohai Strait from late May to late October each year. Furthermore, there were significant interannual and seasonal differences in the number of migratory green lacewings collected. The number of green lacewings in spring was significantly lower than that in summer and autumn, and the highest average number of green lacewings occurred in June. In addition, there were differences in the sex ratio of migrating green lacewings between months, with a greater proportion of females than males. Finally, the seasonal migration trajectories simulated by the HYSPLIT model revealed that the green lacewings captured on Beihuang Island primarily originated from Shandong Province. Accordingly, these findings contribute to our understanding of green lacewing migration in eastern Asia and aid its incorporation within integrated pest management (IPM) packages for several crop pests. Furthermore, long-term tracking of migrant insect populations can reveal ecosystem services and trophic dynamic processes at the macroscale.

## 1. Introduction

Migration is a crucial adaptation strategy for many insect species, allowing them to adjust to environmental heterogeneity and achieve ecological and evolutionary success [1]. In addition to its role in energy and nutrient transfer between regions, insect migration also facilitates the movement of pollen and parasitic microorganisms [2,3]. Both insect herbivores and their natural enemies engage in long-distance migration, and temporal changes in their abundance can mitigate or exacerbate insect-induced crop damage [4,5,6]. Therefore, comprehensive knowledge of the interactions between pests and predators, as well as their long-term migration patterns, is essential for enhancing the integrated control of migratory pests [7]. Natural enemies play a vital role in ecologically based pest control, reducing the reliance on chemical pesticides and providing economic benefits [8]. For example, effective biological control of invasive pests can potentially generate USD 14.6 billion to USD 19.5 billion annually in economic benefit by minimizing yield losses in major food, feed, and fiber crops in Asia [9].

Green lacewings are important natural enemies of insects worldwide. Adults primarily feed on honeydew from homopteran species and floral nectar, and their nymphs are predators of aphids, lepidopteran eggs and early-instar insect larvae, whiteflies, leafhoppers, and many other soft-bodied arthropods [10,11]. There are 1415 lacewing species worldwide [12]. Among these species, 251 species are found in China, including 213 endemic species [13]. Previous studies have shown that green lacewings are the dominant predatory insects in the Bohai Strait, as determined using searchlight trapping [14,15]. There are four known species of migrating green lacewings, namely, *Chrysoperla nipponensis* (Okamoto), *Chrysopa pallens* (Rambur), *Chrysoperla furcifera* (Okamoto), and *Chrysopa formosa* Brauer, and the former two species are the most dominant green lacewing species. Among them, *C. pallens* is dominant in the spring and summer, and *Ch. nipponensis* dominates in autumn. In general, temperature and humidity can have significant effects on the dispersal flight ability of green lacewings. For example, from 15 °C to 30 °C, the migration ability of *C. pallens* and *C. formosa* increased but decreased under high temperature (33 °C) or low humidity (30% RH) [16]. In the UK, green lacewings were found to be the most prevalent species among large insects (>2 mg) captured at a height of 200 meters, second only to highly abundant insects, such as cereal aphids, braconid parasitoids, and small Diptera insects [17]. The migratory populations of *Ch. nipponensis* appear to have adapted to long-distance flights because gender, mating status, and age all determine fight performance [14]. In recent years, several studies have focused on investigating the migration characteristics of important agricultural pest species on Beihuang Island (BH) [14,18,19]. In addition, previous studies have highlighted the significant seasonal and interannual fluctuations in the abundance of certain agriculturally subsidized taxa, revealing the migration of natural enemies alongside noctuid pests [15,20]. Species-specific migration dynamics exhibit pronounced seasonal fluctuations that are potentially influenced by meteorological conditions and the phenology of host plants [21,22]. The migration distance of green lacewings was found to be at least 40–60 km from mainland China, confirming the common occurrence of long-distance migration among green lacewings [23]. Flight mills were used to measure the flight ability of *Ch. nipponensis* for which the maximum cumulative flight distance within 48 h was 43.7 km. Two-day-old individuals who were sexually immature exhibited the strongest flight ability for both females and males. The animals were able to fly for approximately 8 h and covered 20–22 km during the 8 h flight tests. Furthermore, they also had the maximum average flight speed. Mating status significantly affects the flightmill performance of 3-day-old individuals [14]. The common green lacewing *Chrysoperla carnea* (Stephens) occurs during the early reproductive period, and migrations are concentrated in the summer, with some individuals able to fly a distance of approximately 100 km. The flight height in autumn is lower than that in summer and spring. In addition, there is a greater degree of ovarian development in the summer population of green lacewing than in the other populations, but most of the individuals are still in the early stage of spawning, which is conducive to long-distance migration. The ovaries develop quickly to a certain extent, allowing rapid growth of the population after green lacewing reaches its new habitat. In addition, the level of ovarian development in the autumn population of *Ch. nipponensis* was relatively low, which may be related to the shorter duration of sunshine in autumn and the entry of females into reproductive diapause [14]. Therefore, a long-term, systematic assessment of green lacewing migration can help develop and refine strategies to protect or enhance ecosystem services (while reducing disservices, such as pest attacks) within both agricultural and natural habitats.

In recent years, various approaches, including ovarian dissection, light trapping, entomological radar, isotope tracing, and pollen grain analysis, have been used to study insect migration behaviors [14,17,18,21,24,25,26,27]. Entomological radar has been particularly effective at capturing the migration of insect pests in northern China since 2001 [24]. In addition, because massive immigration by predators can potentially provide effective natural control of insect pests, the migratory behavior of these beneficial insects is now receiving increased attention [28]. Suction traps have been widely used for monitoring insect populations, providing accurate measurements of population density and allowing uniform operation under different conditions [29,30]. In the UK, the Rothamsted Experimental Station originally established suction traps to monitor the abundance of aphids and other insects [31], and suction trap monitoring networks have been established in Europe since the 1980s [32,33]. Similarly, in China, suction traps were first installed to monitor wheat and soybean aphids [34,35,36]. A suction trap network provides robust data for analyzing the population dynamics, biodiversity, and bioinformatics of small migrating insects [26,34,35]. To better understand the migration patterns of migratory pests, the Hybrid Single-Particle Lagrangian Integrated Trajectory (HYSPLIT) model was utilized. This model aids in calculating the flight pathways of pests [37,38,39,40,41].

The objectives of this study were to determine the seasonal migration patterns of green lacewings across the Bohai Strait in eastern Asia and the possible migration routes and directions of green lacewing species by simulating backward trajectories and to identify the meteorological factors influencing green lacewing populations using data collected from suction traps over a ten-year period. The findings of this study will contribute to a comprehensive assessment of green lacewing migration behavior and provide valuable insights for biological pest control strategies.

## 2. Materials and Methods

### 2.1. Monitoring of Green Lacewing Species by the Suction Trap on Beihuang Island

**Figure 1:**
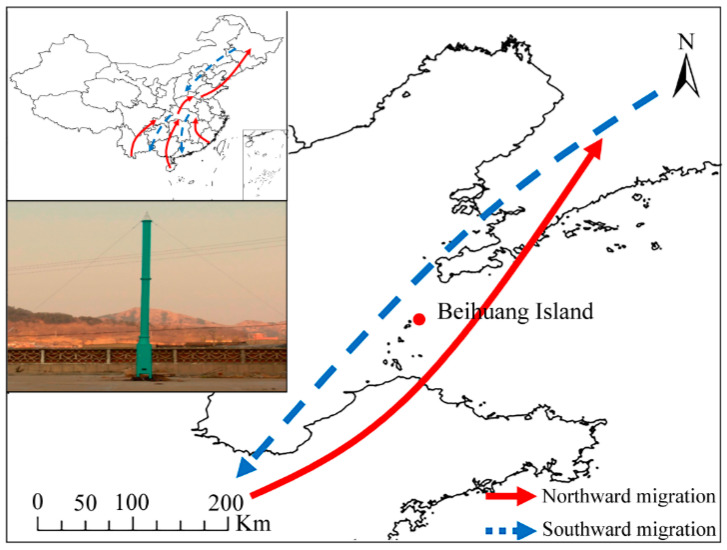
Suction trap on Beihuang lsland (red dot) in eastern China.

The population dynamics of green lacewing species (*Ch. nipponensis*, *Ch. Furcifera*, *C. pallens*, and *C. formosa*) were explored from 2012 to 2021 at the Field Experimental Station for Pest Monitoring and Control, Institute of Plant Protection, Chinese Academy of Agricultural Sciences (Beihuang Island, Shandong Province, 38°23′12″ N, 120°54′30″ E). This location serves as a pathway for the seasonal long-distance migration of many insect species between the northeastern region and the southern, central, and northern regions of China, with immigration occurring from May to June and emigration occurring from August to October. There is no available farmland or farming operations on this island. Northeast China is one of the main crop-producing regions in China, and the BH tract is an important route for many destructive agricultural pests and natural enemies to migrate northwards. Therefore, this island provides an excellent location for monitoring the migration of insects across the Bohai Strait (Figure 1).

A suction trap (ST-1A; Henan Jiyuan Baiyun Industry Co., Ltd., Jiyuan, China) is a device used to capture migrating insects at 12.0 m above the ground. The base of the trap contains a motor and an axial flow fan from which a sample volume of 2000 m^3^ of air per hour is generated. Green lacewings and other insects flying above the trap were drawn through the vertical pipe and collected in a bottle inside the housing. The samples were typically collected every 7 days, although shorter intervals of 3 or 4 days were implemented during peak migration periods from April to October. All the samples were carefully preserved at −20 °C and subsequently stored at the Institute of Plant Protection, Chinese Academy of Agricultural Sciences (Beijing). Species and sex identification was conducted based on the morphological characteristics of the green lacewing species according to the methods of Yang et al. (2023) [42].

### 2.2. Analysis of Main Environmental Factors Affecting the Migration of Green Lacewing Species

The four environmental factors included average weekly temperature, average weekly humidity, average weekly wind speed, and average weekly rainfall, which are important potential basic environmental variables affecting insect migration [41]. The effects of meteorological factors on the capture numbers of the four green lacewing species were analyzed using Canoco 4.5 software (Biometrics, Wageningen, The Netherlands), which revealed the four meteorological factors affecting the capture numbers of the four green lacewing species. To reduce the influence of the bowing effect on the ranking accuracy, the data were processed and analyzed via detrended correspondence analysis (DCA) and length of gradient (LGA). When the LGA is greater than 4.0, canonical correspondence analysis (CCA) is selected; when the LGA is less than 3.0, redundancy analysis (RDA) is selected; and when the LGA is between 3.0 and 4.0, both are used. Meteorological factors were quantitatively analyzed by forward selection and Monte Carlo permutation tests to determine the explanatory strength of meteorological factors on the capture of important migratory pests by suction traps and to test the significance of meteorological variables. Meteorological parameters were downloaded from https://rp5.ru/ (accessed on 4 December 2021) and averaged in the same way as the insect abundance data calculated to determine the effects of different meteorological factors on the capture of different insect species. Thus, the main meteorological factors affecting the capture of migratory pests were identified.

### 2.3. Statistical Analyses

#### 2.3.1. The Abundances of the Four Green Lacewing Species

All samples were processed to calculate the weekly mean values to avoid bias due to variations in monitoring periods. Subsequently, the daily data were summed every 7 days during the first three weeks of each month, resulting in three cumulative units. The fourth unit was calculated by summing the daily means for the remaining days in each month. A total of 28 weekly data points were generated for each species per year. All calculations and analyses were conducted based on 280 weekly data points collected over ten consecutive years. To determine the multiyear and seasonal interactions between green lacewings, we summarized the abundances of the four lacewing species. In addition, the Shapiro-Wilk test was used to assess the normality of all the captured numerical data, while Levene’s test was used to evaluate the homogeneity of variance. After performing a logarithmic transformation of the number of green lacewings captured in different years and months, an analysis of the interannual and seasonal variations in the number of green lacewings captured from 2012 to 2021 was conducted using a general linear model. If the analysis of variance (ANOVA) showed a significant difference (*p* < 0.05), we performed a post hoc analysis using Tukey’s honestly significant difference (Tukey’s HSD) test. Generalized linear mixed-effect Poisson regression (GLM) [43] was used to analyze the raw data for the weekly catches, and this variable number was assumed to be subject to a Poisson distribution. Hence, a corresponding log link function was used. Given that the focus of this study was on seasonal effects, month was considered a fixed effect, while year and year × month were treated as random effects. This mixed-effect regression analysis tested the significance of the fixed effect of the month and the random effect of the year and estimated the weekly number of captured green lacewings for each month from April to October when the effect of that month was significant. The capture numbers of all four lacewing species across different years were analyzed using K-means clustering, with the elbow method used to determine the optimal number of clusters for illustrating variations in yearly capture volumes. All of the above data analyses were carried out using SPSS Statistics 25.0 software (IBM, Armonk, NY, USA).

#### 2.3.2. Sex Ratios of the Four Lacewing Species

The sex distribution of the green lacewings captured by the suction trap each month was analyzed using a chi-square test. To further investigate the variations in the female-to-male ratio among different months and seasons, we conducted a one-way analysis of variance (ANOVA).

#### 2.3.3. Trajectory Simulation

To elucidate the peak migration events of green lacewings, a categorization methodology was applied and defined based on the following set of criteria [44]. These criteria were applied to each instance of green lacewing capture, denoted as N. A week, denoted as week-m, was identified as the peak week if the lacewing captures on that week, N_week-m_, were at least 5 and twice the number of the previous week’s captures, N_week-(m−1)_. If N_week-m_ was a peak date and the following week’s capture occurred, N_week-(m+1)_ was 5 or more, and the following week, week-(m + 1), was also considered a peak week. If the capture on a particular week, N_week-m_, was less than 5 on that week, week-m, it was classified as a nonpeak week. Furthermore, each peak week or a continuous peak period separated by more than 2 nonpeak weeks was considered the beginning of the next peak migration event. The trajectory simulation model HYSPLIT (Hybrid Single-Particle Lagrangian Integrated Trajectory) was used to evaluate the possible migratory sources and migration directions of the species [40,45]. This model utilizes various meteorological data types for its simulations. These datasets include Global Data Assimilation System (GDAS) data, which include global atmospheric data, such as temperature, wind speed and direction, humidity, and pressure at different altitudes. We examined lacewing migration as a point source and analyzed its origin comprehensively, focusing primarily on the impact of air movements, such as wind and rainfall. Data from peak weeks were used to estimate backward trajectories in the migration area. First, due to the small body size of green lacewings, their movement at high altitudes is considered to be the same as the downwind displacement of air particles in this analysis [45,46,47]. Second, the duration of migratory flights of green lacewings can typically reach 12 h [23,48,49]. Third, the migration of green lacewing adults usually occurs at 200–500 m [14,48,49]. Therefore, by running HYSPLIT, 12 and 24 h reverse migration trajectories were calculated for each hour of local time throughout the monitoring season, starting from BH. The simulated migration routes were further analyzed for potential source contribution function (PSCF) analysis using Meteoinfo, which is widely used in the atmospheric sciences to identify potential geographic sources of pollutants [8,50], and the number and percentage of migration trajectory endpoints were counted, marking all possible endpoints as areas where green lacewings were likely to occur.

## 3. Results

### 3.1. The Abundances of the Four Green Lacewing Species

A total of 218 individuals of *C. pallens* were captured from 2012 to 2021. The average annual catch was 21.80 ± 14.82 individuals. The greatest number of *C. pallens* was detected in 2013, with 152 individuals captured. No individuals were captured in 2019 or 2020 (Supplementary Figure S1). One to two migration peaks of *C. pallens* were found on BH in the Bohai Strait of China by observing the number of migrants during these ten years (Supplementary Figure S2). For example, in 2013, there were two migration peaks. One occurred in late June (54 individuals), and the other occurred in early August (47 individuals). In 2016, there was only one migration peak in late June, with 10 individuals. In 2021, there was also only one peak in migration in early July, occurring in six individuals. During the same period, a total of 291 individuals of *Ch. nipponensis* were captured. The average annual catch was 29.10 ± 9.32 individuals. The greatest number of *Ch. nipponensis* individuals were observed in 2017, with 85 individuals captured. There was a significant peak in the migration of *Ch. nipponensis* in early July and late August. The lowest number was recorded in 2012 (6 individuals) (Supplementary Figure S1). The largest number of caught individuals occurred in late September (seven and twenty-seven individuals) in 2015 and 2016, respectively. In 2020, the peaks occurred in early June and late September, with five individuals each. In addition, the numbers of two other species of green lacewings were relatively low compared to those of *Ch. nipponensis*. From 2012 to 2021, a total of 28 individuals of *C. formosa* and 14 individuals of *Ch. furcifera* were captured. The greatest numbers of *C. formosa* were captured in 2013, 2016, and 2021, with thirteen, six, and five individuals, respectively. In contrast, two, seven, four, and one individuals of *Ch. furcifera* were captured in 2015, 2016, 2017, and 2021 (Supplementary Figure S1).

**Figure 2:**
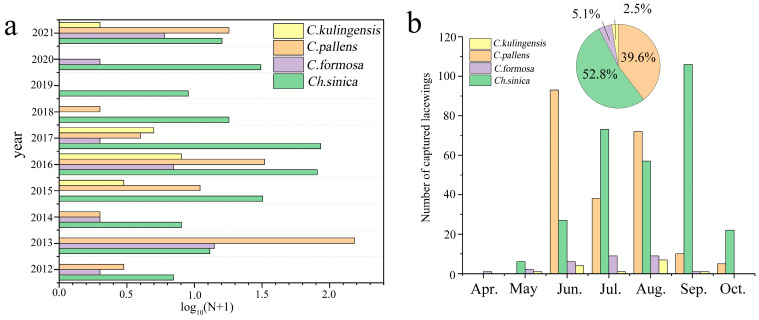
(**a**) The annual capture numbers and (**b**) the monthly capture numbers and percentages of *Chrysoperla nipponensis*, *Chrysoperla furcifera*, *Chrysopa pallens*, and *Chrysopa formosa* via the suction trap on Beihuang Island during 2012–2021.

**Figure 3:**
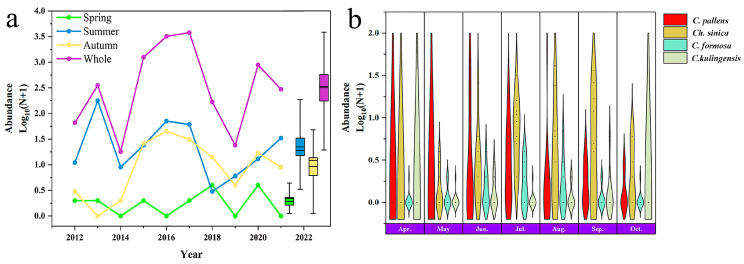
(**a**) Annual migration abundance and (**b**) monthly abundance of the four green lacewing species.

In total, 551 individuals of the four green lacewing species were captured during the decade, with an average annual catch of 55.10 ± 18.27 individuals. The greatest number of green lacewings was captured in 2013, with a total of 177 individuals. Conversely, the lowest number was captured in 2019, with only eight individuals. Among the four species, *Ch. nipponensis* accounted for 53.10% of the total captures, while *Ch. furcifera* accounted for only 2.50% (Figure 2). The number of green lacewings in spring (46.27 ± 30.12) was significantly lower than that in summer (107.98 ± 22.08) and autumn (116.46 ± 57.41) (Figure 3a). The total number of green lacewings also varied significantly among different months (*F*_3, 24_ = 3.70, *p* = 0.03). June had the highest average number of green lacewings (14.70 ± 7.95), which was 146 times greater than the average number in April (0.10 ± 0.10) (Figure 3b).

Overall, the above results showed that the first appearance of the four green lacewing species occurred between early April and late June during these ten years, with the earliest appearance occurring on 28 April 2013. The duration of overseas migration varied, with the longest migration period occurring in 2013 and the shortest occurring in 2019. On average, the duration of overseas migration for green lacewings was 111.80 ± 55.10 days during 2012–2021 (Table 1). Cluster analysis indicated that the total annual green lacewing captures were divided into three groups. In 2019, the lowest number of green lacewings was caught, while in 2013, the greatest number was recorded. The captures in 2016 and 2017 indicated moderate migration, whereas in other years, only weak migration was observed (Supplementary Figure S3). A generalized linear mixed-effect Poisson regression analysis demonstrated that there was no significant interyear variability in the number of green lacewings captured (*Z* = 1.60, *p* = 0.11). In addition, there was a significant month-by-year interaction (*Z* = 2.35, *p* = 0.02) (Table 2).

### 3.2. Sex Ratio of the Four Green Lacewing Species

**Figure 4:**
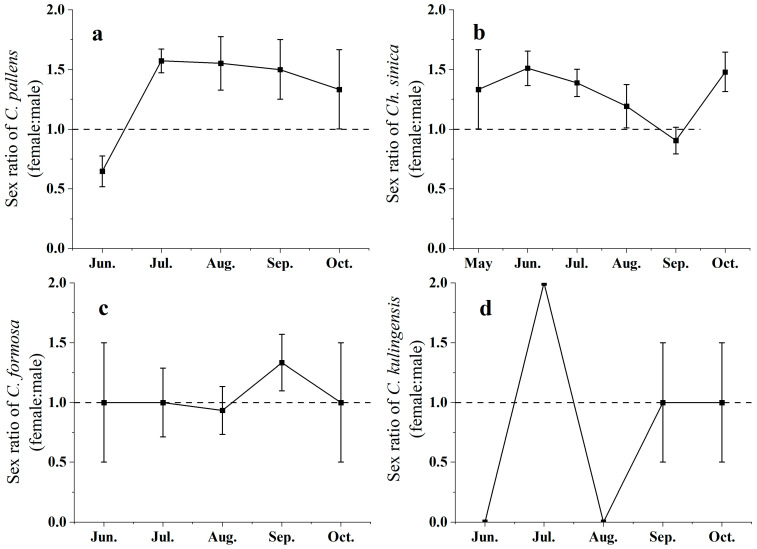
Seasonal variation in the proportions (Mean ± SE) of (**a**) *Chrysopa pallens*, (**b**) *Chrysoperla nipponensis*, (**c**) *Chrysopa formosa*, and (**d**) *Chrysopa furcifera* females captured by the suction trap during 2012–2021.

Overall, the sex ratio of green lacewings varied among different months, with more females being captured than males. The proportion of females also exhibited significant seasonal differences (*F*_10,25_ = 8.74, *p* < 0.05) (Figure 4). More female green lacewings than male green lacewings were captured in July. In contrast, in other months, the difference in the number of males and females was not significant (May: χ^2^ = 0.54, *df* = 1, *p* > 0.05; June: χ^2^ = 0.98, *df* = 1, *p* > 0.05; August: χ^2^ = 3.40, *df* = 1, *p* > 0.05; September: χ^2^ = 2.40, *df* = 1, *p* > 0.05; October: χ^2^ = 2.24, *df* = 1, *p* > 0.05). Specifically, the sex ratios of *C. pallens* were 32.34% ± 12.88% (June), 78.63% ± 9.93% (July), 77.53% ± 22.46% (August), 75.00% ± 25.00% (September), and 66.66% ± 33.33% (October) from June to September. In contrast, from May to October, the sex ratios of *Ch. nipponensis* were 66.66% ± 33.33% (May), 75.51% ± 14.44% (June), 69.36% ± 11.47% (July), 59.56% ± 18.21% (August), 45.25% ± 11.10% (September), and 74.00% ± 16.61% (October) (Supplementary Figure S4).

### 3.3. Main Environmental Factors Affecting the Migration of the Four Green Lacewing Species

**Figure 5:**
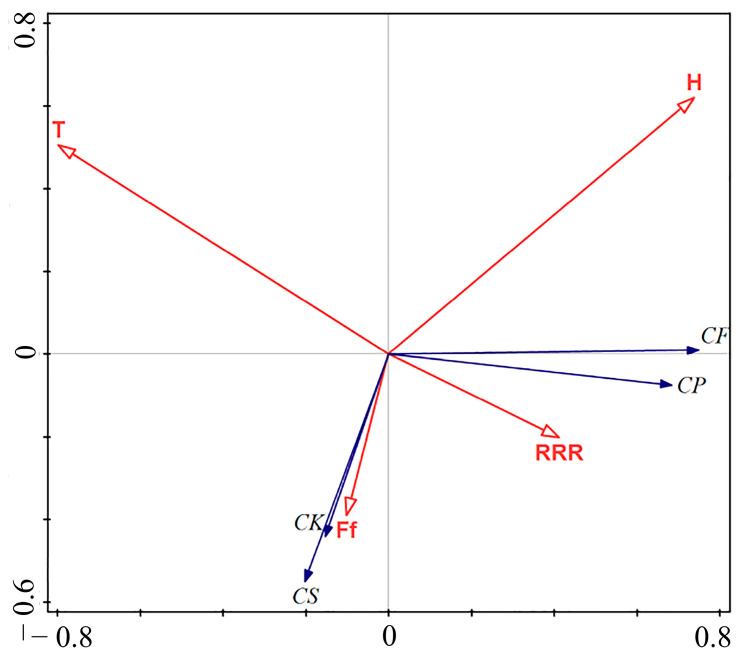
RDA biplots of the capture numbers of the four green lacewings and meteorological factors on Beihuang Island from 2012 to 2021. The smaller the angle between the vectors (or a vector and an axis) and the longer the vectors are, the more correlated the variables represented by the vectors are. All the environmental variables (with significant (solid arrows) effects on the number of green lacewings captured) are shown. Note: CS: *Chrysoperla nipponensis*; CK: *Chrysopa furcifera;* CF: *Chrysopa formosa*; CP: *Chrysopa pallens*; T: average weekly temperature; H: average weekly humidity; Ff: average weekly wind speed; RRR: average weekly rainfall.

Overall, the redundancy analysis (RDA) results revealed that four meteorological factors accounted for 46.06% of the variance in migration numbers in the four species of green lacewings, and the average weekly temperature, average weekly humidity, average weekly wind speed, and average weekly rainfall exhibited no significant correlations with green lacewing migration number or abundance variability, contributing 24.80%, 14.20%, 4.20%, and 2.90%, respectively. Notably, the catch numbers of *Ch. nipponensis* and *Ch. furcifera* exhibited a strong correlation with wind speed, while the catch numbers of *C. pallens* and *C. formosa* were primarily affected by the average weekly rainfall. The numbers of the four green lacewings showed a weak correlation with the average weekly temperature and the average weekly humidity (Figure 5). The mean daily temperature in 2016 had a significant impact on the number of green lacewings (2016: *F* = 4.40, *p* < 0.05) (Supplementary Table S1).

### 3.4. Potential Migration Pathways of the Two Dominant Lacewing Species

**Figure 6:**
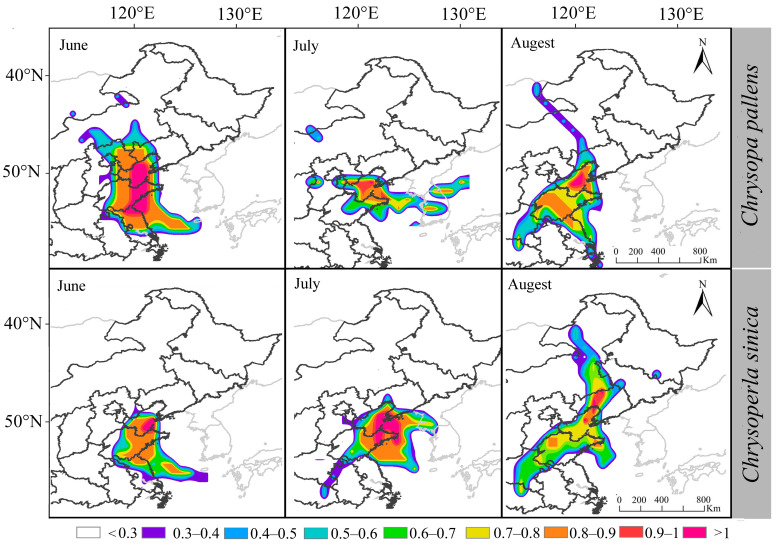
Possible source locations for two dominant species (*Chrysoperla nipponensis* and *Chrysopa pallens*) captured on Beihuang Island in different months. The different colors indicate the likelihood of the PSCF analysis results for migrating through the landing site.

To understand the migration patterns of the four green lacewing species, we analyzed the numbers of the two dominant species (*Ch. nipponensis* and *C. pallens*) trapped on BH from 2012 to 2021. Specifically, we focused on the typical migration events that occurred from June to September. Using the HYSPLIT model, we conducted a forward trajectory analysis of the migration paths considering both 12 h and 24 h intervals. The results indicated that the sources of trapped green lacewings on BH varied by month. For *C. pallens*, the main source of migratory flights in June was Shandong (59.36%). In July, the main sources shifted, with Shandong (47.22%) and Hebei (25.22%) located south of Beihuang Island being the most significant. In August, Shandong (59.82%) remained a major source, while Russia (15.38%) and Jiangsu (12.50%), located north or south of Beihuang Island, became other sources. Similarly, for *Ch. nipponensis*, the main sources of migratory flights in June were Shandong (65.28%) and Hebei (19.44%). In July, Shandong (56.79%) remained a major source, along with Liaoning (10.93%), which is located north of Beihuang Island. In August, it was 37.81% in Shandong and 25.63% in Liaoning. In September, it was 46.19% in Shandong and 32.62% in Hebei. Overall, Shandong Province emerged as the primary source for both green lacewing species during the peak period of green lacewing migration (Figure 6).

## 4. Discussion

### 4.1. Interannual and Seasonal Variation in the Abundance of Four Green Lacewing Species

Long-distance migration prevents adverse environmental conditions, such as extreme climate conditions, food shortages, predation, and other pressures, from occurring to find a more suitable breeding site, which plays a vital role in the life history of many insects. Beihuang Island is positioned within one prime insect migration corridor that has a unique setting for assessing insect populations across space and time and is an ideal location for observing insect migration. Previous studies have focused mainly on the migration characteristics of several important agricultural migratory pest species on BH [1,51,52]. However, knowledge of the trans-sea migration of natural enemies in eastern Asia is still limited [53,54,55,56]. In this study, a suction trap was used to monitor the natural enemies of four green lacewing species on the island, and the results confirmed that these species can migrate at least 40–60 km from mainland China during the migration process. Therefore, long-distance migration is a common phenomenon in the population ecology of green lacewing species. Long-distance migration contributes to a variety of benefits for green lacewings, allowing them to take advantage of spatially unevenly distributed resources, such as food and habitat, which can improve their nutritional status. Significant variations were observed in the number of green lacewings captured on BH across different months and years, which aligns with findings from investigations on other agricultural pests [51]. This difference may be attributed to differences in the migratory behavior of different green lacewing species, including their migratory height and migratory behavior rhythm. In general, there are overall differences in insect biology between *Chrysopa* (both adults and larvae are predaceous, with a narrow range of prey groups, mainly aphids, and the overwintering stage is a prepupa) and *Chrysoperla* (only larvae are predaceous, with a large range of prey groups, and the overwintering stage is an adult). In Hubei Province, the pupae of *C. pallens* overwinter inside their cocoons and begin to emerge in mid-April, with the peak period occurring from late April to early May. In contrast, in the northern regions of China, the population of *Ch. nipponensis* is rare before June and is primarily found in orchards and wheat fields, and its abundance increases after July with the occurrence of pests. These differences may be related to differences in individual development, temperature adaptability, and feeding habits between the two species of lacewings [57]. *C. nipponensis*, *C. pallens*, and *C. formosa* are widely distributed in the northern Yangtze River. Even in northern or southern China, there are differences in the dominant species among different regions. These three species are the dominant species in the Aksu region, Xinjiang, and the Loess Plateau of Shaanxi Province [58]. The flight ability of green lacewings is also influenced by other factors, including age, mating status, host food availability, and environmental and climate conditions. For instance, only immature *Chrysopa cornea* adults can migrate long distances, which is a very short period—the first couple of days after emergence. These immature adults fly at much greater heights above the ground (above 5 m) than in later phases of their development and thus are dispersed mostly by wind (downwind migration), which is an obligatory migration flight [48,49]. *Ch. nipponensis* is most capable of flying 2 to 3 days after emergence. The flight ability of unmated 3-day-old individuals was greater than that of mated individuals [14]. This species has a long egg-laying period of approximately one week, which not only provides sufficient time for its migration but also ensures that it has enough energy and materials for use during its migration [59]. Notably, we discovered a decrease in the total number of captured green lacewings over the past decade. For instance, the greatest number of green lacewings was recorded in 2013 (177 individuals), while the lowest was observed in 2019 (eight individuals). Similarly, as the number of captured natural enemies in BH declined over the past decade, the predatory bugs *Cyrtorhinus lividipennis* (Reuter) [60] and *Pantala flavesces* [55] were also observed via searchlight trapping. This phenomenon might be due to the loss of habitat, agrochemical pollution, or irreversible changes in the microclimate at the natal and stopover sites [55,61,62]. Further analysis revealed that the species composition of the green lacewings varied among the different months, with *Ch. nipponensis* and *C. pallens* being the dominant species. The migration peak of *C. pallens* was observed in June and August, while the greatest number of migrating *Ch. nipponensis* occurred in July and September. These variations in abundance were attributed to differences in reproductive cycles and ecological preferences between the two species. Both *C. pallens* and *C. formosa* exhibit predatory behavior during the adult stage and prey on pests to meet their nutritional needs during mating [63]. In contrast, *Ch. nipponensis* generally does not exhibit predatory behavior during its adult stage [63,64]. Furthermore, *Ch. nipponensis* has been found to exhibit long-distance migratory flight before its reproductive system matures [49].

The large-scale migration of multiple insect species is an important ecological phenomenon that highlights the interactions between different trophic levels, including predators, hosts, and plants, suggesting the potential for coevolution between functional groups [7,15]. Many predators, including lacewings, ladybugs, hoverflies, and carabid beetles, as well as some parasites, often migrate together with the pests upon which they prey [59]. Natural enemies tend to migrate either simultaneously or slightly behind the pests they feed on [7,41,62,65]. The lagged migration of natural enemies can signal an evolutionary adaptation through which the prior establishment of pests provides sufficient food resources and promotes the successful colonization of natural enemy populations [21,65]. In the future, we will focus on large-scale comigration and interactions between green lacewings and pests, which can effectively control pest populations [15].

### 4.2. The Sex Ratio of the Four Green Lacewing Species

Our findings indicate that there were more females of the four green lacewing species than male green lacewings captured, which is consistent with the findings of several studies [66]. Female green lacewings might tend to undergo long-distance migrations to maximize their reproductive potential. In general, when insects migrate to areas abundant in food, their nutritional status can increase, and their fertility can increase, consequently enhancing their environmental adaptability [67]. Previous studies have indicated that more than 50% of the migratory population is female, most of which are in the preoviposition period and have low degrees of ovarian development [14]. In general, individuals capable of green lacewings for long-distance migration are immature adults, which is an obligatory process, and only these individuals fly at high heights above the ground. Similarly, our results also indicated that more female green lacewings than male green lacewings were captured in July. A greater proportion of *C. pallens* females were in July and September, as well as *Ch.* There was a greater proportion of *N. nipponensis* females in June and October. This pattern is consistent with the dominance of young females with underdeveloped ovaries in migratory green lacewing populations, which is considered an adaptation to long-distance migration [14,48]. In addition, female green lacewings may require sufficient food to provide the nutrients necessary for reproduction, and the migration of lacewings could be the result of long-term evolutionary selection [68,69]. If the migratory behaviors of green lacewings lead to reproductive success, then females inclined to migrate will pass this behavioral tendency to the next generation [70].

In addition, a previous study showed that most females in the migratory population of *Ch. nipponensis* were in the preoviposition period, which indicated that reproduction lagged behind migration. The ovarian development of most females reached the third level for summer migrants of *C. nipponensis*, while it reached the first level for most females of autumn migrants. Therefore, the indices of ovarian development were greater in summer than in autumn [14]. In contrast, the proportion of females captured in September was the lowest among those captured using light traps during 2008–2009. Considering that this study was conducted over a relatively short period of only two years, it may be influenced by several factors, including collection methods and climatic conditions [14,71]. Therefore, in the future, obtaining data from additional years and increased numbers of diverse green lacewing species will be more accurate to elucidate the sex ratio of the four green lacewing species.

### 4.3. Meteorological Factors Affecting the Number of Captured Green Lacewings

Long-distance insect migration is carried out by the wind load of the seasonal upper air current, and long-distance insect migration is related to seasonal variations in the wind pattern. The dispersal of major crop pests in China is also accelerated by the stepwise movement of the East Asian summer monsoon, which generates long-term southwesterly/southerly air currents that penetrate major cropping regions throughout East China [72]. Consequently, monsoon-driven dispersal further exacerbates the difficulties in monitoring and forecasting Chinese crop pests.

A previous study indicated that humidity, wind speed, precipitation, and temperature were significantly correlated with the numbers of two rice planthopper species, *Laodelphax striatellus* (Fallén) and *Sogatella furcifera* (Horváth), and five natural enemies [41]. However, at present, there is no research on the relationships between meteorological factors and *Chrysoperla* spp. The flight ability of the green lacewing *Ch. nipponensis* did not significantly differ between the sexes at different relative temperatures (18–28 °C) or relative humidities (45–75%) [14]. *Chrysoperla* species generally prefer temperatures ranging from 20 to 30 °C and humidities ranging from 50 to 80%, although overall, they exhibit relatively strong environmental adaptability [73]. Normally, green lacewings belonging to the genus *Chrysoperla* exit diapause at an average temperature of 10 °C, initiating their migration from overwintering locations to diverse habitats. They feed on pollen and nectar while engaging in predatory behaviors and proceed with their breeding activities [74]. In this study, the results indicated that average weekly temperature, average weekly humidity, average weekly wind speed, and average weekly rainfall exhibited no significant correlations with green lacewing migration numbers or abundance variability. Similarly, studies of the effects of different habitat types on the suitability of lacewings have indicated that landscape configuration, such as elevation, wind speed, humidity, temperature, and rainfall, has no significant impact on the number of green lacewings in Turkey [75]. Temperature and humidity also had no significant impact on the number of *Chrysoperla* spp. in vineyards within landscapes in southwestern France [76]. However, the overall abundance of the green lacewing community increased with increasing temperature [77]. In addition, migratory lacewings mainly flew at altitudes less than 300 m during the whole flight season. In autumn, lacewings flew at altitudes less than 100 m, which was lower than their flight heights in spring and summer. The dense insect layers were significantly and positively related to the altitudes of maximum temperature and low-level maximum wind speed, respectively, during the peak migration nights of lacewings. The flight duration of the migratory lacewings was approximately 9 h. The displacement speeds were 24.63, 24.33, and 27.26 km/h for the spring, summer, and autumn migrants, respectively, which were all significantly related to the wind speed. The displacement directions were strongly related to the downwind directions. In spring and summer, lacewings commonly originated in northern China and moved toward the northwest or northeast to exploit new habitat patches, while their offspring massively migrated back south to overwintering sites in early autumn. Green lacewings could change their displacement directions slightly by adjusting the angles between their orientation directions and downwind directions when the winds were unfavorable. Notably, the catch numbers of *Ch. nipponensis* and *Ch. furcifera* exhibited the strongest correlation with wind speed, while the catch numbers of *C. pallens* and *C. formosa* were primarily affected by the average daily rainfall, which is consistent with the findings of previous studies in the region [78]. Considering that the capture site was on an island, this could be the reason why humidity was more significant for the results of this study.

### 4.4. Green Lacewing Species Originating Mainly in Northeastern, Northern, and Eastern China

The long-distance migration of insects is linked to seasonal changes in climate, seasonal upper air currents, resource availability, and wind patterns. China is located in the East Asian monsoon zone, and the East Asian monsoon is conducive to large-scale insect migration. Specifically, the flat terrain around the Bohai Strait serves as a crucial corridor for insect migration in East Asia [14,19,79,80,81]. Trajectory analysis is a powerful tool for tracing the origin and predicting the populations of migratory insects [40,45,67,81,82]. In this study, it was found that green lacewings primarily migrated from Shandong Province for most months, which is consistent with the findings of other studies [59,81]. Based on the migration numbers of lacewings collected by high-altitude searchlights from 2017 to 2021, tracing analysis was carried out with BH as the endpoint. The source of lacewings on BH showed that in spring, the lacewings mainly came from North China, mainly from Shandong, Henan, and Jiangsu, and a small part came from merging and Liaoning. The migration direction was mainly from south to north. The summer lacewings were mainly from Shandong and Liaoning Provinces, and there were both northward- and southward-migrating populations, which exhibited north-south two-way mixed migration. In autumn, lacewings mainly come from Liaoning, Hebei, Shandong, and other provinces, indicating that there is still a phenomenon of lacewings migrating north in autumn [59]. The HYSPLIT model is a meteorological analysis platform for tracing and predicting the transfer of inorganic particles, but the ability to migrate insects was not included in the model, which might have led to a short trajectory distance and orientation deviations. Therefore, the accuracy of trajectory analyses depends on the reasonable setting of flight parameters, such as time, speed, and altitude. In this study, due to the small number of green lacewings captured by the suction trap and the different effects of monsoons on the migration of green lacewings in different years and seasons, we cannot accurately determine their southwards and northwards migration. At present, due to the influence of collection accuracy, we can only preliminarily obtain the trend and change in the two dominant green lacewings, *Ch. nipponensis* and *C. pallens*. Based on real-time aerial behavior parameters of migrating insects, an integrated entomological radar trajectory analysis model and other technologies will improve the accuracy of trajectory and forecasting [41]. We will establish additional monitoring sites, including several possible landing points obtained from our trajectory analysis. Moreover, methods of ovary dissection could be used to further study green lacewings captured by suction traps during different periods and at different collection sites to confirm their migration paths and migration patterns.

### 4.5. Pest Management Implications

Natural enemies play a vital role in biological control services in both natural and man-made environments and are a fundamental aspect of integrated pest management [56,83]. The effectiveness of natural enemies in pest control depends on the synchronized colonization of functional groups within the agricultural landscape [84] and is influenced by their characteristics and habitat preferences [85]. To gain a more precise understanding of green lacewing migration patterns, future research can incorporate additional methods, such as insecticide resistance monitoring, insect isotope tracing, and genetic diversity analysis. Exploring other aspects of green lacewing migration, such as timing, distance, ovarian development, mating frequency, and sex ratio, can provide a more comprehensive understanding of this topic. Further research is needed to fully understand the adaptations associated with long-distance migration, including factors such as morphology, hormone levels, development time, growth rate, and distribution of energy stores. Expanding and enhancing monitoring networks will provide valuable scientific data on the population dynamics, bioinformatics, and biodiversity of other migratory insects, complementing our understanding of green lacewing migration.

## Figures and Tables

**Table 1:** Migration periods and abundances of four green lacewing species captured by suction traps on Beihuang Island in northern China during 2012–2021.

Year	Date of First Capture ^a^				Date of Final Capture ^a^				Migration Duration (Days)			
	*Ch. nipponensis*	*C. pallens*	*C.* *formosa*	*Ch. furcifera*	*Ch. nipponensis*	*C. pallens*	*C.* *formosa*	*Ch. furcifera*	*Ch. nipponensis*	*C. pallens*	*C.* *formosa*	*Ch. furcifera*
2012	14-July (2)	29-May (1)	20-June (1)	—	—	17-July (1)	—	—	1	49	1	0
2013	23-June (10)	30-June (5)	28-April (1)	—	19-August (22)	4-August (6)	19-August (1)	—	57	35	113	0
2014	1-October (1)	16-June (1)	25-June (1)	—	—	1-October (5)	—	—	1	107	1	0
2015	7-July (2)	7-July (7)	—	25-August (1)	13-October (2)	13-October (1)	—	8-September (1)	98	98	0	14
2016	13-June (8)	13-June (1)	30-May (1)	6-June (1)	10-October (1)	24-October (2)	8-August (1)	15-August (2)	119	133	70	70
2017	19-June (1)	5-June (9)	24-July (1)	29-May (1)	11-September (1)	2-October (5)	—	21-August (1)	84	119	1	84
2018	17-September (1)	27-May. (1)	—	—	—	24-September (1)	—	—	1	120	0	0
2019	—	6-July (3)	—	—	—	19-September (1)	—	—	0	75	0	0
2020	—	28-May (2)	28-May (1)	—	—	30-October (1)	—	—	0	155	1	0
2021	3-June (2)	3-July (1)	18-July (2)	3-June (1)	29-September (2)	9-October (1)	29-September (1)	—	118	98	73	1

^a^ Amounts of green lacewings captured are given in parentheses next to the name of the month.

**Table 2:** Summary of the generalized linear mixed-effects regression. The target is the number of green lacewings caught per week by the suction trap. The fixed effect and random effects are month and year (year × month), respectively.

Fixed Effect	Test	Factor	*F*	*df_1_*	*df_2_*	*p*-Value	LB	UB
		Month	2.41	9	97	0		
	Estimate	Level	Coef	SE	*t*	*p*-Value		
		Intercept	1.55	0.310	5.01	0	0.94	2.17
		April	−2.87	1.18	−2.44	0.02	−5.21	−0.53
		May	−1.10	0.47	−2.33	0.02	−2.02	−0.16
		June	−0.40	0.33	−1.22	0.23	−1.05	0.25
		July	−0.38	0.30	−1.28	0.21	−0.97	0.21
		August	0.07	0.35	0.21	0.84	−0.63	0.77
		October	1.55	0.31	5.01	0.00	0.94	2.17
		September	0.00 ^a^					
Random effect	Test	Factor	Variance	SE	*Z*	*p*	LB	UB
		Year	0.44	0.28	1.60	0.11	0.13	1.50
		Month × Year	0.25	0.11	2.35	0.02	0.11	0.57

LB and UB: lower and upper bounds of the 95% confidence interval, respectively. ^a^ Redundant parameter.

## Data Availability

Data are contained within the article.

## Supplementary Materials

The following supporting information can be downloaded at https://www.mdpi.com/article/10.3390/insects15050321/s1. Figure S1. Population dynamics of four species of green lacewingsbythesuction trap during 2012–2021; Figure S2. Monthly average of four green lacewings captured by the suction trap during 2012–2021; Figure S3. Clustering dendrogram of the lacewings captured by the suction trapduring 2012–2021.; Figure S4. Seasonal variation in the sex ratio of (a) *Chrysopa pallens*, (b) *Chrysoperla sinica*, (c) *Chrysopa formosa*, and (d) *Chrysopa kulingensis* females captured by the suction trap during 2012–2021. Table S1. The explained variation inmeteorological factors and results of significancetests of four lacewing species onBeihuang Island during 2012–2021.
