# Supplementary material for: Abundance and Seasonal Migration Patterns of Green Lacewings (Neuroptera: Chrysopidae) across the Bohai Strait in Eastern Asia
Source: Insects. 2024 May 1;15(5):321. doi: 10.3390/insects15050321 (PMC11121799; doi:10.3390/insects15050321)
Supplement: Supplementary file 1 [file insects-15-00321-s001.zip › insects-2829510-supplementary.pdf]

Abundance and Seasonal Migration Patterns of Green Lacewings (Neuroptera: Chrysopidae) across the Bohai Strait in Eastern Asia

Xingya Wang [1], Haotian Ma [1], Yuechao Zhao [1], Ying Gao [1] and Kongming Wu [2,*]

[1] College of Plant Protection, Shenyang Agricultural University, Shenyang 110866, China; wangxingya20081@163.com (X.W.); mhtpony@163.com (H.M.); zhaoyuechao98512@163.com (Y.Z.); gy434585296@163.com (Y.G.)
[2] State Key Laboratory for Biology of Plant Diseases and Insect Pests, Institute of Plant Protection, Chinese Academy of Agricultural Sciences, Beijing 100193, China
* Correspondence: wukongming@caas.cn

Supporting Information

Figure Legends

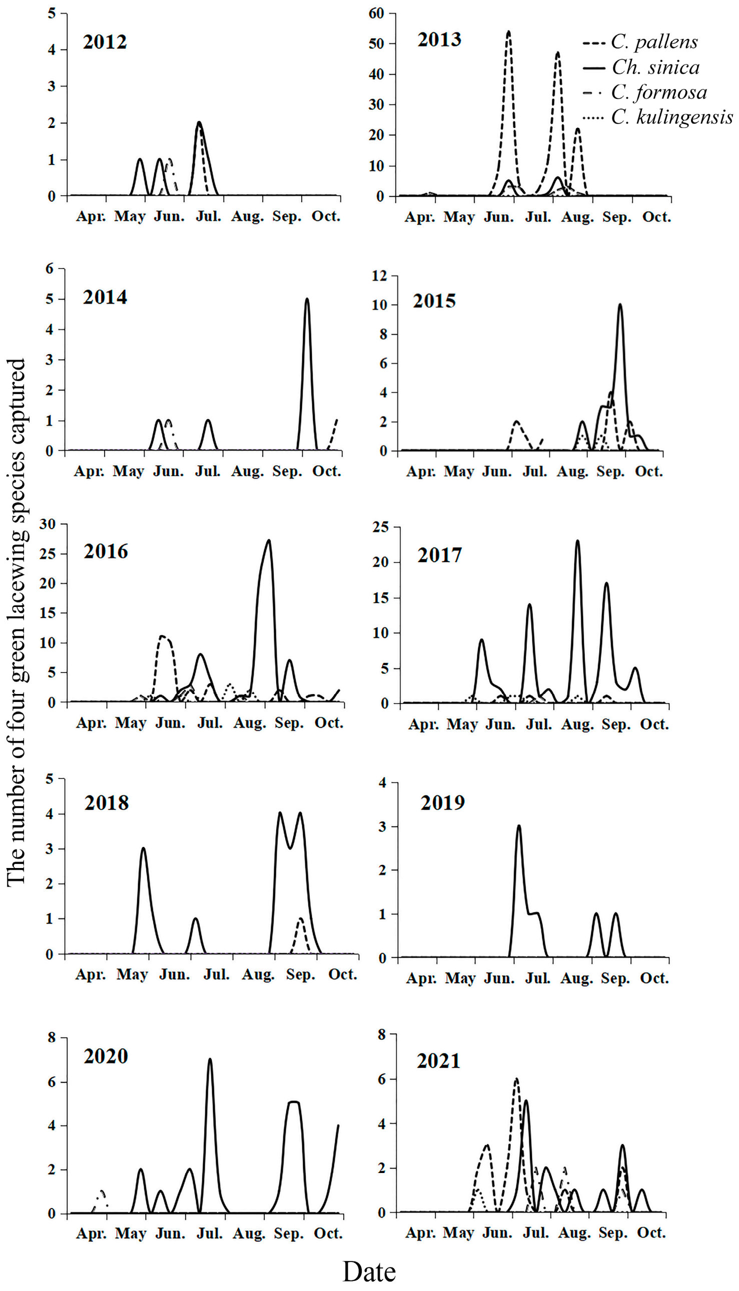

Supplementary Figure S1. Population dynamics of four species of green lacewings by the suction trap during 2012-2021.

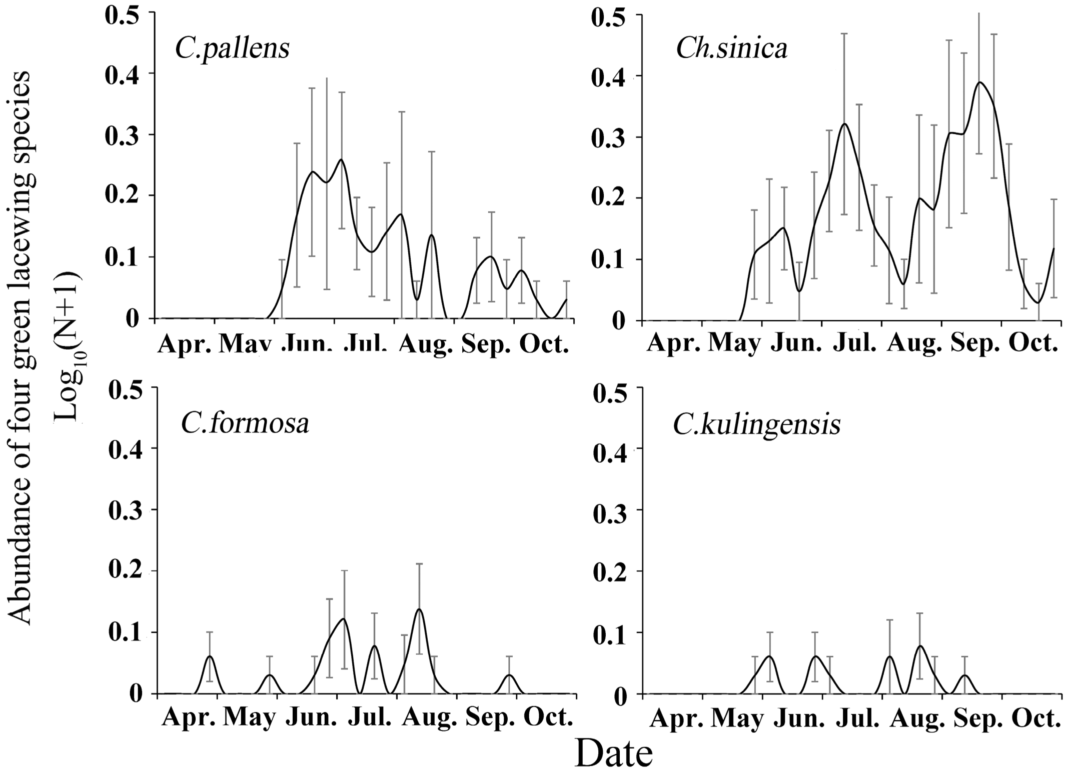

Supplementary Figure S2. Monthly average of four green lacewings captured by the suction trap during 2012-2021.

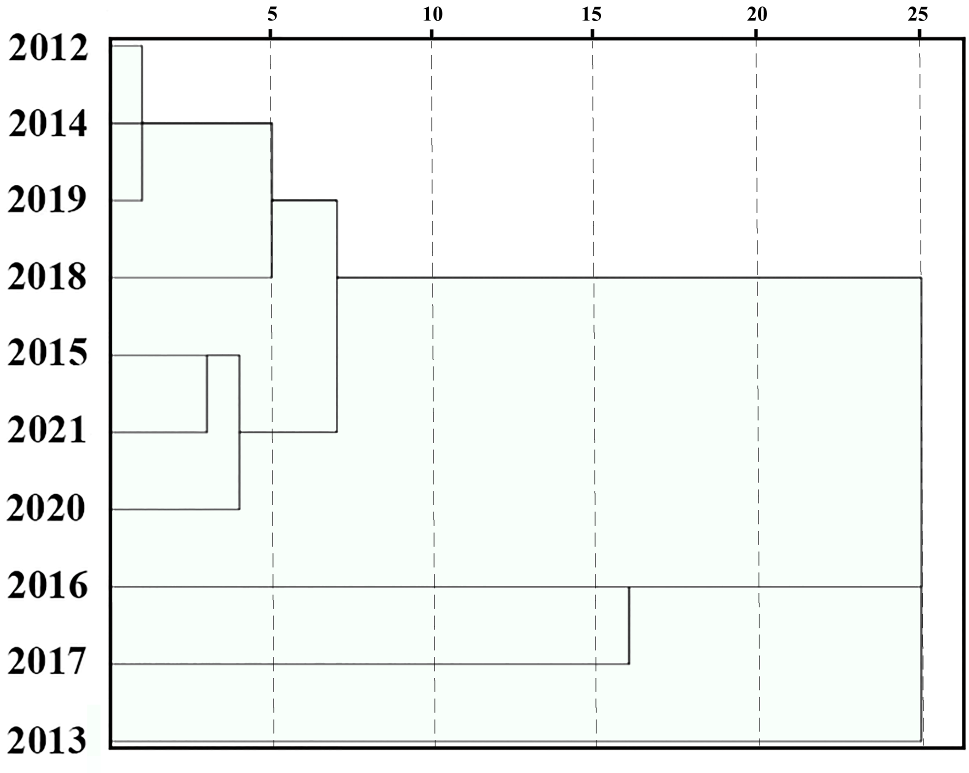

Supplementary Figure S3. Clustering dendrogram of the lacewings captured by the suction trap during 2012-2021.

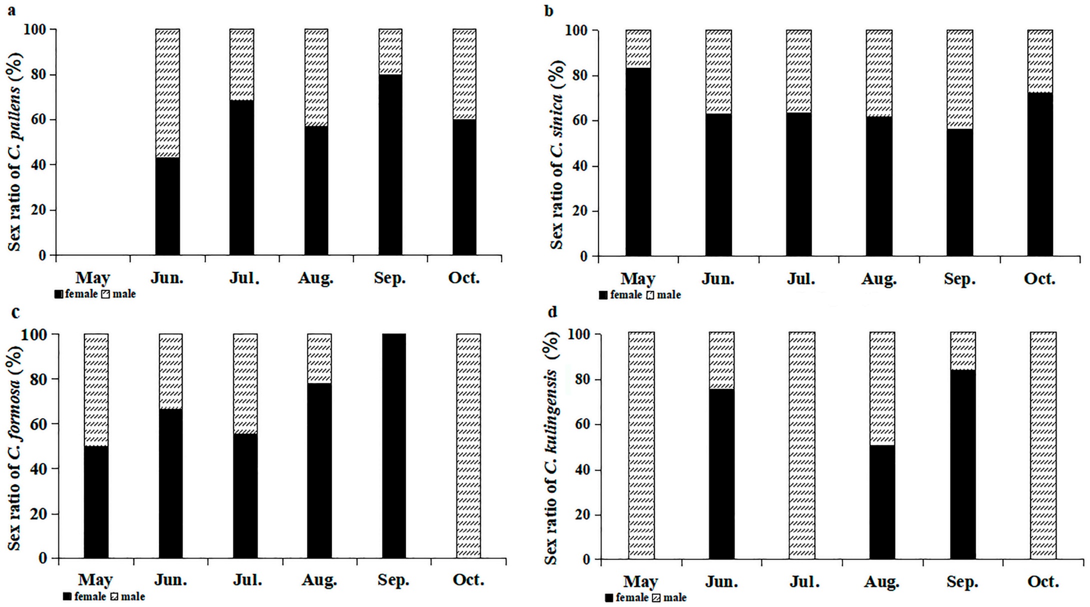

Supplementary Figure S4. Seasonal variation in the sex ratio of (a) *Chrysopa pallens*, (b) *Chrysoperla sinica*, (c) *Chrysopa formosa*, and (d) *Chrysopa kulingensis* females captured by the suction trap during 2012-2021.

Supplementary Table S1. The explained variation in meteorological factors and results of significance tests of four lacewing species on Beihuang Island during 2012-2021.

Meteorology factors	Year	Variance Explains (%)	Meteorological factors contribution rate (%)	Pseudo-*F*	*P*
	2012	0.5	8.8	0.1	0.954
	2013	1.2	5.4	0.3	0.708
	2014	2.3	8.2	0.4	0.548
	2015	1.5	14.8	0.4	0.562
Average daily humidity	2016	2.7	11.4	0.9	0.442
	2017	4.1	21.9	0.6	0.634
	2018	0.7	21.3	0.2	0.678
	2019	3.0	30.5	0.7	0.420
	2020	0.5	6.1	0.1	0.752

	2021	6.3	41.0	1.6	0.212
	2012	1.7	29.3	0.4	0.666
	2013	2.4	10.5	0.6	0.506
	2014	5.5	20.0	1.1	0.380
	2015	0.9	8.7	0.2	0.662
Average daily wind speed	2016	2.1	9.0	0.7	0.494
	2017	5.8	30.8	0.9	0.460
	2018	0.4	13.4	0.1	0.800
	2019	0.7	6.9	0.2	0.880
	2020	5.3	69.2	1.4	0.260
	2021	1.3	8.3	0.3	0.776
	2012	1.6	26.8	0.4	0.700
	2013	10.2	45.6	2.6	0.092

	2014	16.9	61.6	3.5	0.086
	2015	5.4	52.5	1.4	0.238
Average daily temperature	2016	13.5	56.6	4.4	0.016*
	2017	6.4	34.3	1.0	0.430
	2018	1.8	53.2	0.4	0.534
	2019	3.1	31.5	0.8	0.416
	2020	0.4	5.6	0.1	0.768
	2021	5.6	36.2	1.4	0.256
	2012	2	35.1	0.5	0.560
	2013	8.6	38.5	2.1	0.092
	2014	2.8	10.2	0.6	0.478
	2015	2.5	24.0	0.7	0.438
Average daily rainfall	2016	5.5	23.0	1.8	0.168

	2017	2.4	13.0	0.3	0.770
	2018	0.4	12.1	0.1	0.792
	2019	3.1	31.2	0.8	0.362
	2020	0.5	6.1	0.1	0.752
	2021	2.2	14.5	0.5	0.622
